# Neighbourhood Influences on Population Health: Time to Unpack the Black Box

**DOI:** 10.3389/phrs.2022.1605053

**Published:** 2022-06-08

**Authors:** Ana Isabel Ribeiro, Pedro Gullón, Emily T. Murray

**Affiliations:** ^1^ EPIUnit—Instituto de Saúde Pública, Universidade do Porto, Porto, Portugal; ^2^ Laboratório para a Investigação Integrativa e Translacional em Saúde Populacional (ITR), Porto, Portugal; ^3^ Departamento de Ciências da Saúde Pública e Forenses e Educação Médica, Faculdade de Medicina, Universidade do Porto, Porto, Portugal; ^4^ Public Health and Epidemiology Research Group, School of Medicine, Universidad de Alcalá, Alcalá de Henares, Spain; ^5^ Centre for Urban Research, RMIT University, Melbourne, VIC, Australia; ^6^ Department of Epidemiology & Public Health, University College London, London, United Kingdom

**Keywords:** neighborhood effect, environmental health, urban health, geographic information systems, inequalities

Modern neighborhoods and health research is now over 30 years old [1]. After a period of childish curiosity guided by naïve methodologies and a puberty period of trying to fit in, neighborhoods and health research has matured into a highly competitive research area of public health and epidemiology. And yet, this is just the beginning. A long path until full maturity is still to be paved.

Mounting evidence suggests that individuals who live in places with underprivileged social and physical environments are at higher risk of different health conditions and mortality [2]. However, there are many missing links in the current evidence base, related to study theorization, causal inference, exposure assessment, and translation into evidence-based interventions.

In the same way biomedical experiments explicitly outline and test the molecular and genetic pathways that connect a given causal agent to a given outcome, a comparable approach should be sought when investigating neighbourhood effects. This calls for well-formulated theories and frameworks backed up by biological, environmental, and social sciences. Certainly, there are many well-conceptualized theoretical perspectives; see, for instance, Kieger’s “ecosocial theory of health” conceived to bond health, biology, sociopolitical and environmental histories under a multilevel framework [3]; Pearce’s “life-course of place approach” designed to understand the coevolution of people and places and their relationships over time [4]; or Macintyre’s “deprivation amplification model” developed to explain the interdependency between individual and neighbourhood socioeconomic conditions [5].

Using the hierarchy of evidence pyramid as a standard to rank epidemiological designs, it becomes obvious that weak study design is an integral part of the causal inference problem in neighborhood effects research. A 2016 review of US research found that over 70% of the studies were cross-sectional [6]. Using cohort studies and incorporating the life-course framework allows researchers to overcome the implausible assumption of simultaneity of effects underlying cross-sectional designs, while helping to understand if the accumulation of place-based exposures over life, early life exposures, and critical and sensitive exposure periods contribute to the interpretation of neighbourhood effects on health. Though, longitudinal cohort design is not a synonym of strong causal evidence. In fact, very few studies have used longitudinal data and proper panel-study methods to examine within-person health changes due to moves or modifications in the neighbourhood environment. Quasi-experimental studies are underrepresented in the literature too. Nonetheless, they provide a clear temporal ordering of causes and effects and much-appreciated information for policy-makers on the effectiveness of place-based interventions.

Neighbourhood and health research has also been plagued by systematic errors such as residual confounding (when omitted variables result in non-exchangeability of individuals across neighborhoods) and residential self-selection bias (when people choose their residential locations because of their attitudes, likings, or social inequity). Despite the pervasiveness of these challenges, methods to account for such biases are underused and underdeveloped, and most research uses multiple regression; in detriment of more promising methods like propensity score matching, marginal structural models, population restriction, or instrumental variables [7, 8].

Additionally, neighbourhood exposures are typically only assessed in conveniently available, static and farfetched areas, such as census/statistical units. Consequently, results might be sensitive to the shape of the spatial units or the scale of analysis, and/or might fail to characterize the multitude of “activity spaces” which people are exposed to in their daily life, as well as migration flows (populations are becoming increasingly nomadic). Methodologies such as GPS, geotagged social media, geographic momentary assessment or social network analyses may lead to better specified contextual measures.

Furthermore, although frameworks such as the exposome—the totality of environmental exposures from conception onward—have been preached, these have not been truly incorporated in health and place research. The exposome can provide a holistic framework to investigate interactive relationships between diverse multi-scalar exposures, their epigenetic effects, and long-term health consequences [9]. Yet, incorporating high resolution, decades-long exposure data is challenging to say the least—data protection regulation related to geoprivacy is increasingly strict, certain exposures were not measured in the past, and available datasets often use incompatible formats.

Finally, research on place-based interventions is scarce and fairly new. Inclusively, we lack evidence on the dual effects of certain public health interventions on health inequalities. For example, urban regeneration may impel gentrification processes which can lead to upgrades in environmental quality, improving health among stayers; but, may also cause forced displacement, generating stress and residential insecurity. Furthermore, while many translation models exist—some driven by research evidence, others based on community inputs—these models have been poorly documented using case studies, and most public health practitioners and researchers lack the capacities necessary to translate knowledge into evidence-based interventions.

In conclusion, health and neighbourhoods research can generate information of remarkable public health relevance but it can also be held by a number of shortcomings like insufficient theorization, oversimplistic exposures or unideal study designs. Thus, this research topic aims to collect evidence syntheses and evidence-informed methods and recommendations to overcome the obstacles that challenge health and place research.
